# Relationship between burnout syndrome and age among employees of the Mexican manufacturing industry

**DOI:** 10.3389/fpsyg.2025.1542497

**Published:** 2025-06-04

**Authors:** Aide Aracely Maldonado-Macías, Mónica Gabriela Gutiérrez-Hernández, Manuel Alejandro Barajas-Bustillos, Yordán Rodríguez, Arturo Realyvásquez-Vargas

**Affiliations:** ^1^Departament of Industrial Engineering and Manufacturing, Autonomous University of Ciudad Juarez, Chihuahua, Mexico; ^2^Department of Industrial and Logistics Engineering, National Technological Institute of Mexico/Technological Institute of Ciudad Juarez, Chihuahua, Mexico; ^3^National School of Public Health, University of Antioquia, Medellín, Colombia; ^4^Department of Industrial Engineering, National Technological Institute of Mexico/Technological Institute of Tijuana, Mexico

**Keywords:** burnout syndrome, age, manufacturing industry, validation, relationship

## Abstract

**Introduction:**

Nowadays, burnout syndrome (BS) has been widely studied due to the increasing and high work demands to which workers are exposed. To date, there is a lack of studies that analyze the relationship between age and its impact on employees in the Mexican manufacturing industry.

**Objective:**

This research aims to determine the relationship between BS, its dimensions, and age among operative employees, senior, and middle managers in this industry.

**Methods:**

The 16-item Maslach Burnout Inventory-General Survey (MBI-GS) was used to gather data and measure BS by grade and level. Nine hundred thirty-three employees voluntarily answered the instrument. First, the instrument was validated through psychometric tests to ensure reliability. Kaiser-Meyer-Olkin (KMO) and sphericity tests confirm the feasibility of the factorial analysis. The model’s fit adjustment was tested using structural equation modeling with AMOS. Then, the BS was obtained by grades and levels. Subsequently, the Spearman Rho correlation analysis was carried out between the BS grades by dimension (Emotional exhaustion, Cynicism, and Lack of Professional Efficiency) and age group (up to 40 years and over 40 years). Finally, a hypothesis test for differences between means was conducted to determine whether there were significant differences by BS dimension’s grade regarding age.

**Results:**

The instrument’s reliability is good, with Cronbach’s alpha value greater than 0.8 for each dimension. The instrument’s structure was confirmed through exploratory factor analysis (EFA). However, the structural equation models do not meet the goodness-of-fit criteria when the sample is divided into two age groups. Furthermore, the entire sample obtained medium grades for each dimension and a medium level of BS. Significant but weak correlations were obtained between age, cynicism, and professional efficiency. The mean test shows significant differences in the dimensions of cynicism (*P*−*value* 0.004) and professional efficiency (*P*-*value* 0.003). Likewise, the Tukey test with α = 0.05 revealed significant differences in these dimensions between the 51 and 60 age group and the 21-30 and 31-40 age groups.

**Conclusion:**

The sample studied has a medium BS level. Accordingly, age exhibits negative and positive correlations with cynicism and professional efficiency, respectively, even when they are weak. Psychometric tests corroborate the instrument’s reliability, not its validity.

## 1 Introduction

Burnout syndrome (BS) or occupational exhaustion BS is considered a neuropsychological disorder resulting from chronic stress at work (Arji et al., 2023). It is a widespread, multifactorial, and psychological phenomenon (Listopad et al., 2021). Additionally, according to the World Health Organization, BS is considered an occupational phenomenon, not a medical condition (World Health Organization, n.d).

BS is a phenomenon with significant consequences at both personal and professional levels. Several studies have explored its effects in different contexts, highlighting its impact on mental health, academic performance, and job stability. Other studies in the academic context found that BS affects medical students, leading to feelings of cynicism and inadequacy. Collaboration and peer learning can help mitigate these effects, but BS remains a significant problem affecting students’ performance and mental health (Gómez et al., 2024).

In the workplace, BS can increase employee turnover intentions. However, contextual factors often mediate the mechanisms linking burnout to these outcomes. A study conducted at the Portuguese Tax and Customs Authority found that occupational stress and BS are significantly associated with intentions to leave (Freitas et al., 2023). However, this relationship may be weaker in organizations with robust support systems, suggesting that structural interventions could mitigate turnover. Employees who experience high stress levels and BS tend to look for other jobs due to a lack of motivation and emotional exhaustion. Additionally, studies among healthcare professionals have shown that BS has even more serious consequences since a study in Portugal revealed that BS is related to an increase in suicidal behaviors (de Jesus et al., 2023). These authors informed that self-esteem could moderate these effects, but the risk of suicidal behaviors remains high when BS is severe. In addition, toxic leadership can exacerbate BS, increasing employee turnover intentions. Emotional intelligence can moderate this relationship, helping employees better manage the stress and negative emotions associated with BS (Lopes et al., 2025).

Consequently, recent studies have found that BS can affect students, employees of various professions, and job positions of various ages at multiple levels or grades.

Conventionally, BS is measured by emotional exhaustion, depersonalization or cynicism, and reduced professional efficacy. The first dimension can manifest as feelings of being overextended. Depersonalization/cynicism refers to cynical and negative attitudes toward work and the people with whom one interacts (e.g., patients and coworkers), and reduced professional efficacy is associated with poor work performance (Maslach et al., 2001). However, new evidence has emerged suggesting that this phenomenon may be more extensive, and therefore, the three dimensions may not fully capture its complex and multifaceted nature (Listopad et al., 2021). In addition, several sociodemographic variables such as gender (Marchand et al., 2018), marital status (Armenta et al., 2021), schooling (Norlund et al., 2010), daily working hours (Macias-Velasquez et al., 2020), seniority (Korman et al., 2022), among others, have been related to the presence of BS in employees of a wide variety of professions.

Still, the relationship between age and BS has been uncertain in industrial employees, mainly middle and senior managers; while some authors affirm that BS rates increase with age (Marchand et al., 2018; Arji et al., 2023), other authors affirm that this relationship can be both (Rožman et al., 2017, 2018, 2019a), and other authors reveal a moderating effect (van der Westhuizen et al., 2015). Employees in these job positions are critical for the accomplishment of the companies’ objectives, supporting drastic changes for improvement by facilitating communication and information among other managers or subordinates (Abdullah and Sofyan, 2022), but they are also more vulnerable to BS effects (Ahola et al., 2010; Rožman et al., 2019b). Furthermore, several factors, including age, work involvement, and coworker cohesion, have been linked to BS dimensions among industrial workers. Specifically, work pressure is linked to emotional exhaustion and depersonalization, while professional accomplishment is related to job satisfaction (Lam et al., 2022).

As established previously, part of the problem is that the relationship between age and BS remains undefined in the literature. In addition, several studies in the Mexican manufacturing industry context have determined the prevalence of this syndrome among their employees (Maldonado-Macías et al., 2015; Macias-Velasquez et al., 2020; Armenta-Hernández et al., 2021). These authors address these gaps by focusing on Mexican industrial managers, a population underrepresented in the literature, while controlling for moderators like work hours and marital status. However, the relationship between age and burnout remains unclear. This research aims to determine the relationship, dimensions, and age among frontline employees and senior and middle managers in the industry.

## 2 Literature review

### 2.1 Maslach burnout inventory-GS grades and levels

As verified in the literature, BS can be measured along three dimensions. Additionally, five levels of BS derived from combinations of these three dimensions (Emotional Exhaustion, Cynicism, and lack of Professional Efficacy) can provide a framework to identify the intensity of BS and guide interventions. These levels can be interpreted based on established norms and cut-offs and are usually labeled as follows (Bakker et al., 2002; Schaufeli et al., 2010; Wang et al., 2024):

1. *No BS*: This level indicates that the individual is functioning well with no signs of BS, including low exhaustion, low cynicism, and high professional efficacy.
2. *Mild BS*: Moderate exhaustion and/or cynicism accompanied by sustained professional efficacy. This is a warning stage at which some symptoms of BS may begin to appear.
3. *Moderate BS*: This involves increased exhaustion and cynicism, accompanied by potential declines in professional effectiveness. Individuals may feel detached and experience increasing stress.
4. *Severe BS*: Characterized by high exhaustion, cynicism, and significant reductions in professional efficacy. Individuals at this level are at a critical juncture where their work performance and personal well-being are severely impacted.
5. *Critical BS*: Extreme exhaustion, cynicism, and low professional efficacy. As BS is deeply entrenched at this stage, individuals may require urgent interventions.

### 2.2 Relationship between age and Maslach burnout

A search of three databases (IEEE, ScienceDirect, ACM) by keyword found that few studies present evidence of a relationship between age and BS among industry workers.

Among these works analyzed two groups of workers in Saudi Arabia: one under 40 and the other 41 and older. His study found no significant differences between the two groups regarding stress, BS, or age. BS is more significant among civilian government workers in China’s Shandong province who are under 36 years old. These results align with those reported analyzed a sample of 444 workers in a service area in Germany. Their findings suggest that age indirectly predicts less BS and greater commitment through emotional regulation strategies, superficial performance, and anticipatory deep performance. In the case of the work presented by Hatch et al. (2018), a sample of 400 nursing workers in a health system in the southeastern United States found that older workers may be better able to take advantage of cognitive, psychological, and work strengths that improve with age.

Additionally, in a study carried out with 9,922 people, of whom 7,765 were of working age, BS does not increase or decrease constantly concerning age but probably decreases and increases with age, taking as a differentiator the various phases of the work career and family life since today, employees do not remain in the same situation throughout their working careers. Although some positions may be more likely for BS during specific years, such as becoming a parent during the first few years of work or a manager during later years, transitions can affect the accumulation of the total burden in each age group (Ahola et al., 2008). Additionally, Rožman reports diverse results from three studies conducted on various samples of workers in Slovakia. In two studies, BS is found to have a negative relationship with age (Rožman et al., 2017, 2019a), whereas in another study, it is positive (Rožman et al., 2017).

In the industrial and service sectors, the relationship between BS and age remains poorly understood (Marchand et al., 2018). For example, in the results mentioned that female workers under 25 exhibit higher rates of BS compared to other age ranges, as observed in a study conducted among 1,081 female workers in Guangdong, China. In contrast, Marchand et al. (2018), found that BS presents a positive relationship concerning age in an age group of workers under 30; subsequently, the relationship becomes negative until 55, and at the end, it becomes positive again, this in a sample of 2,073 workers in the manufacturing and service industry in the province of Quebec, Canada.

In contrast, the work published by van der Westhuizen et al. (2015), which used a sample of 246 workers from an international integrated energy and chemicals company in South Africa, found no statistically significant evidence to support differences between age categories. However, they found they could be used as predictors in some dimensions. According to other studies, older workers exhibit better mental stability due to their experience, unlike younger workers, who often experience higher levels of stress and family conflicts. Younger workers may still need to learn to adapt to the work environment, while older workers may be more resilient in adjusting to a changing environment (Hsu, 2019; Jurek, 2024).

Therefore, some other authors have found that these variations may stem from:

1. Cultural differences: For instance, younger workers in collectivist cultures (e.g., China) may experience higher burnout due to familial pressures absent in individualist contexts.
2. Career stages: Transitions (e.g., parenthood, promotions) may temporarily spike burnout, confounding age effects (Ahola et al., 2008).
3. Methodological limitations: Cross-sectional designs (Macias-Velasquez et al., 2020) cannot disentangle age from cohort effects.

## 3 Materials and methods

### 3.1 Study design

This research employs a quantitative approach, featuring a cross-sectional design and correlational scope (Hernández et al., 2014). It aims to investigate how age affects BS among Ciudad Juárez manufacturing industry workers. The Institutional Committee of Ethics and Bioethics of the Autonomous University of Ciudad Juarez endorsed this research on October 22, 2019 (Folio: CIEB-2019-1-098).

### 3.2 Participants

The sample comprises 933 participants who voluntarily completed the instrument after signing the informed consent: 92 managers, 236 supervisors, 242 technicians, 105 group leaders, and 258 frontline employees. The exhibition features various industries, including medical products, automotive, electronics, and miscellaneous sectors, located in Ciudad Juárez, Chihuahua, Mexico. The study was conducted from November 2019 to November 2021.

### 3.3 Instrument

The Spanish version of the Maslach Burnout Inventory-General (MBI-GS) was used for data collection, having been translated and adapted by Moreno-Jimenez et al. (2001). This instrument contemplates three dimensions. The first dimension is emotional exhaustion, which involves feelings of exhaustion resulting from the high physical and mental demands of work. It is indeed considered an obvious manifestation of the syndrome. On the other hand, the dimension of cynicism identifies when the person tries to distance himself from the service recipient. It is presented as a negative response to various aspects of work, where the worker only wants to do their job without being involved in additional activities. Finally, the dimension of reduced professional efficiency is considered a self-evaluation. Address feelings of incompetence and lack of work productivity (Maslach et al., 2001).

The instrument consists of 16 items grouped into three dimensions: emotional exhaustion, professional efficacy, and cynicism. They are seven-point Likert scales where 0 = never, 1 = rare throughout the year, 2 = sometimes throughout the year, 3 = on many occasions throughout the year, 4 = frequently throughout the year, 5 = almost every day, and 6 = every day. Of the 16 items, 10 have a negative meaning, and only six are positive (see Table 1).

**TABLE 1:** Dimensions of the Maslach BS inventory-general survey instrument.

Dimension	Item code	Feeling that represents
Emotional exhaustion	1. Totally exhausted	Exhausted emotionally
	2. Exhausted	Finishing at the end of the day
	3. Fatigued	Fatigued at dawn
	4. Stressed	Work is stressful
	6. Exhausted	Exhausted by my work
Professional efficiency	5. Resolve	Able to solve problems
	7. Contribution	Contribution to the work
	10. Good	I am good at doing my job.
	11. Carried out	I feel fulfilled
	12. Value	Realized worthwhile things
	16. Efficacy	Effective in doing my job
Cynicism	8. Interest	Loss of interest
	9. Enthusiasm	Loss of enthusiasm
	13. Bothered	Do not bother me
	14. Indifferent	I have become indifferent.
	15.Doubt	I doubt the value of my work

Adapted from Moreno-Jimenez et al. (2001).

### 3.4 Methods

**FIGURE 1:**
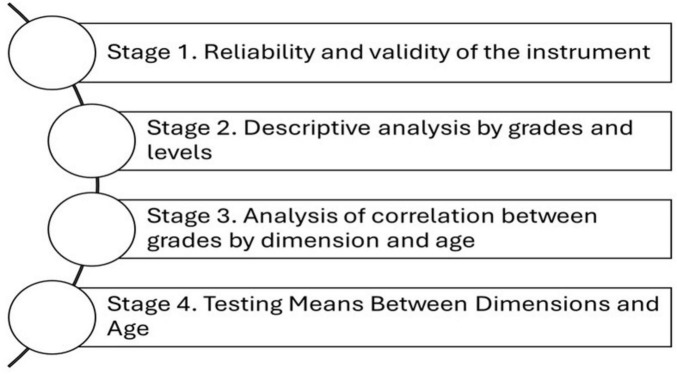
Stages of the methodology. Source: The authors.

The study was developed through four stages (see Figure 1). The objective was to determine the degrees and levels of BS among workers in the manufacturing industry. Subsequently, the type of relationship between age and the presence of the syndrome was determined. Additionally, the reliability and validity of the instrument used were confirmed. In this context, studies such as those by Macias-Velasquez et al. (2019) and Armenta-Hernández et al. (2021) served as a reference for the analysis and interpretation of the results obtained from the instrument. Refer to Hair et al.’s multivariate data analysis book for the psychometric tests.

#### 3.4.1 Stage 1. Reliability and validity of the instrument in the sample

In research, it is essential to corroborate the reliability and validity of the instrument. On the other hand, reliability corresponds to the precision with which the variable studied is measured. Among the most used indicators to measure it is Cronbach’s alpha, obtained through the correlations between the items that comprise the instrument. Values greater than 0.70 correspond to an acceptable level of reliability (Bland and Altman, 1997; Tavakol and Dennick, 2011; Rodríguez-Rodríguez and Reguant-Álvarez, 2020). In addition to evaluating the instrument’s reliability with the complete sample, the instrument was divided into two groups, using the age of 40 years as a reference. The first group is up to 40, while the second group includes workers over 40.

Once the instrument’s reliability is confirmed, it is necessary to perform the construct validation to ensure that it measures what it intends to measure. Factor analysis is recognized as the ideal technique to guarantee the viability of the sample and the validation of the construct (Pérez-Gil et al., 2000). However, the relevance of the factor analysis must be verified first. For this purpose, the indicators used were the determinant of the correlation matrix, the Bartlett’s sphericity test, and the Kaiser-Meyer-Olkin index (KMO).

Subsequently, it is recommended to start with exploratory factor analysis (EFA) to confirm that the items were grouped into the correct dimensions according to the structure proposed by Moreno-Jimenez et al. (2001). Factorial extraction was performed using the principal component method. Factors with a self-value greater than one were excluded. The initial matrix was rotated using the Varimax orthogonal method, which provides the most obvious factorial separation (Hair et al., 2013) and shows little likelihood of producing inadequate solutions (Fabrigar et al., 1999; Stevens, 2012).

The construct validation was performed through confirmatory factor analysis when these procedures were conducted. This analysis was performed using structural equation modeling in the statistical software AMOS^®^ version 22. This analysis aimed to confirm that the instrument’s dimensions comply with the statistical parameters (Byrne, 2016). The maximum likelihood method was used to calculate the factor loads. The parameters used to evaluate the absolute fit of the model were the chi-square statistic (χ^2^), the chi-square ratio concerning the degrees of freedom (χ^2^/df), the root-mean-square error of approximation (RMSEA), the goodness-of-fit (GFI), and the adjusted goodness-of-fit statistic (AGFI). On the other hand, the Normed fit index (NFI), the Comparative fit index (CFI), and the Tucker–Lewis index (TLI) were used to evaluate incremental fit. The AFC was performed on the entire sample (933 participants) and age-related groups. The first sample corresponds to participants up to 40 years old, and the second corresponds to participants over 40 (308 participants).

#### 3.4.2 Stage 2. Descriptive analysis by grades and levels

The database was created, cleaned, and registered on June 29, 2022, and its registration number is 03-2022-062712044100-01 according to the National Institute of Authorship Rights in Mexico (INDAUTOR). The first step was to divide the sums of each dimension’s responses and use the 33.3 and 66.6th percentiles as cut-off points to determine the degree of BS (low, medium, high). Subsequently, the mean scores by dimension were calculated and used to determine the grade for each dimension according to the values presented in Table 1. Finally, the level of BS is a categorical variable of five categories (none, low, moderate, high, and extremely high). These levels are obtained from the 27 possible combinations derived from the three degrees of BS. The statistical analysis was performed using SPSS version 24 software.

#### 3.4.3 Stage 3. Analysis of the correlation between grades by dimension and age

Because the data are ordinal and we wanted to know the relationship between the dimensions of BS (emotional exhaustion, cynicism, and reduced professional efficiency) and age (taking 40 years as a reference), Spearman’s Rho was used as a correlation index. This is a non-parametric measure of correlation, through which the relationship between two variables is described.

#### 3.4.4 Stage 4. Testing means between dimensions and age

The Tukey test is used to identify precisely where the means (compared to each other) are different from the rest (Benites, 2022). This test was performed at this stage to identify the differences between the BS’s grades according to six age range groups (under 21, 21-30, 31-40, 41-50, 51-60, and over 60).

## 4 Results

### 4.1 Sample characteristics

The sample comprises 626 men and 307 women working in manufacturing companies. The most frequent age range was 31-40. In addition, various positions and company sectors were considered, with the medical products sector obtaining the highest number of responses (see Table 2).

**TABLE 2:** Sample description.

Age		Job position	
**Under 21 (years old)**	11	Manager	92
**21-30**	291	Supervisor	236
**31-40**	323	Technical	242
**41-50**	234	Group leader	105
**51-60**	69	Frontline employee	258
**More than 60**	5		
**Company sector**			
**Electronics**	67	Automotive	318
**Medical products**	397	Miscellaneous	151

Source, the authors.

#### 4.1.1 Stage 1 Results. Reliability and validity of the instrument in the sample

##### 4.1.1.1 Reliability

Cronbach’s alpha corroborated the instrument’s reliability. Overall reliability was 0.856, while by dimension, it was emotional exhaustion (0.878), cynicism (0.811), and professional efficiency (0.941). Likewise, when analyzing the reliability of the groups, values higher than 0.80 were obtained in both groups. The first group was 0.868, and the second was 0.831. Values greater than 0.8 refer to good reliability (Bland and Altman, 1997; Rodríguez-Rodríguez and Reguant-Álvarez, 2020).

##### 4.1.1.2 Construct validity

Subsequently, the feasibility of factor analysis was verified through the three minimum indicators: the determinant, Kaiser-Meyer-Olkin (KMO), and Bartlett’s sphericity test. Statistical analysis was performed using SPSS version 26 software. Table 3 shows the results that corroborate the feasibility of factor analysis (Kaiser and Rice, 1974; García-Castellanos et al., 2020).

**TABLE 3:** Relevant indicators for the EFA.

Indicator	Value	Reference
Determinant	0.00001061	Value ≠ 0
Bartlett’s sphericity test	10603.866 120 DF *p* = 0.000	*p*-value < 0.05
Kaiser–Meyer–Olkin (KMO)	0.897	Value > 0.7

Source: The authors.

On the other hand, in the exploratory factor analysis, three factors were extracted, which represent 70.723% of the total variance explained. The distribution of the items in these factors confirms the structure proposed by Moreno-Jimenez et al. (2001).

Only items with a value equal to or greater than 0.4 were considered to determine their influence on the factor. Table 4 shows the arrangement of the items and the resulting loads of each item. Likewise, commonalities allow us to know how each item correlates with others. This represents the amount of deviation explained in terms of each item, and values higher than 0.1 are recommended (Barrera Ortiz et al., 2015).

**TABLE 4:** Rotated matrix of components and communalities.

#	Item description	Factor			Communalities
		1	2	3	
1	I feel emotionally drained by my work.		0.794		0.730
2	I feel finished at the end of the workday.		0.895		0.826
3	I feel fatigued when I wake up in the morning and must start another day of work		0.867		0.743
4	Working all day is stressful for me.		0.825		0.700
6	I feel finished with my work		0.651		0.596
5	I can effectively solve problems that arise in my work	0.785			0.691
7	I feel that I am making an effective contribution to the activity of my organization	0.830			0.702
10	In my opinion, I am very good at doing my job	0.924			0.823
11	I feel fulfilled when I accomplish something in my work	0.906			0.850
12	I have done a lot of worthwhile things in my work	0.914			0.823
16	In my work, I am sure that I am effective at doing things	0.905			0.744
8	Since I started the job, I have been losing interest in my work			0.788	0.669
9	I have been losing enthusiasm for my work			0.802	0.676
13	I want to do my job and not be bothered			0.522	0.363
14	I have become more indifferent about whether my work is worth anything			0.834	0.363
15	I doubt the value of my work			0.866	0.706
	Extraction method: principal component analysis. Rotation method: Promax with Kaiser normalization.				
	To. The rotation has converged in 5 iterations.				

Source: The authors.

Once the instrument’s structure has been verified through the EFA, the construct validity was evaluated by modeling structural equations. AMOS version 22 software was used (Byrne, 2016). Table 5 shows the results obtained. For the model that includes the entire sample (*n* = 933), it is observed that it complies with two of the absolute adjustment parameters, the Root Mean Square Error Approximation (RMSEA) and the Goodness-of-fit Index (GFI). The (RMSEA) represents the anticipated adjustment with the population’s value and not only the sample. The GFI determines how much of the variance and covariance of the sample is explained by the model.

**TABLE 5:** Structural confirmatory model.

Index	Sample values			Recommended
	Complete	Up to 40 years old	Over 40 years old	
**Model fit**				
Chi-square of the estimated model *X*^2^	499.766 DF = 95 Value *p* = 0.000	687.955 df = 94 Value *p* = 0.000	400.89 Shadow Fiend = 100 Value *p* = 0.000	*p* > 0.05
*x*^2^/DF	5.261	7.319	**4.009**	<5
**Absolute fit measures**				
RMSEA	**0.068**	0.101	0.099	≤ value 0.08
90% RMSEA CI	(0.062; 0.074)	(0.094; 0.108)	(0.089; 0.109)	
GFI	**0.908**	−	0.836	>0.90
AGFI	0.868	−	0.777	>0.90
**Incremental fit measures**				
TLI	0.788	0.899	0.712	>0.90
NFI	0.803	**0.910**	0.708	>0.90
CFI	0.832	**0.921**	0.760	>0.95

The numbers in bold refer to those values that comply with the reference values.

On the other hand, when separating the sample into two groups, up to and over 40 years of age, a considerable change in the parameters is observed. Referring to the model, those up to 40 years old do not comply with any absolute adjustment parameters. However, it complies with incremental fit parameters, which measure the degree to which the model’s fit is improved compared to the hypothetical model. These parameters are regularly used to explain the models better since absolute indicators such as the chi-square are highly dependent on the sample size (Schmukle and Hardt, 2005) and are challenging to comply with. Furthermore, the incremental parameters, such as Tucker–Lewis’s (TLI) and the non-normed fit index (NNFI), compare the model studied against the hypothetical model. Commonly, this indicator performs best in small samples (Byrne, 2016) and is usually compared against 0.9 to indicate an acceptable fit (McDonald and Ho, 2002). In this study, a marginal value of 0.899 is nearly acceptable. Likewise, the model achieved the Normed fit index (NFI). The first analyzes the improvement of the fit by comparing the studied model against the hypothetical model (Hooper et al., 2008); the second compares the chi-square of the model studied against the hypothetical one. For both parameters, a value greater than 0.9 is considered acceptable (Gefen et al., 2011). Finally, the model with participants over 40 years of age only complies with one absolute fit parameter: the chi-square ratio for degrees of freedom, with a value less than 5.

#### 4.1.2 Stage 2. Descriptive analysis by grades and levels

When analyzing the qualities of the Emotional Exhaustion (EE) dimension, it shows us a score with ranges from 0 to 30 points, with an arithmetic mean of 7.8574 and a standard deviation of 5.95801. The trend on the cynicism (CC) dimension takes us to a range of 0-28 points, with an arithmetic mean of 7.0214 and a standard deviation of 5.57158. While in the professional efficiency (PE) dimension, a minimum range of 1 to 36 points is observed, with an arithmetic mean of 23.0043 and a standard deviation of 9.38358. Likewise, Table 6 shows that the analyzed sample reports a medium degree for the three dimensions.

**TABLE 6:** Distribution of scores by dimension.

Dimension	Emotional exhaustion EE	Cynicism CC	Professional efficiency PE
Low	*5* points	*4* points	*18* points
Medium	6–9 points **(7.86)**	5-9 points **(7.02)**	19–29 points **(23.00)**
High	10points	10points	30points

The values in bold correspond to the average scores per dimension.

**FIGURE 2:**
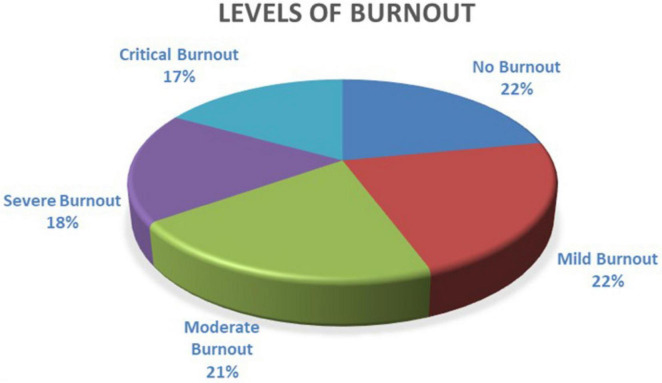
Levels of BS. Source: The authors.

On the other hand, when analyzing the levels of BS, the sample is similarly, distributed in the five levels (see Figure 2). However, 56 % of the sample presents levels of the syndrome ranging from moderate to critical. Employees experiencing these levels of BS may present adverse symptoms such as being detached and continuously more stressed, reducing performance at work, affecting personal wellbeing, and requiring urgent interventions (Maslach and Jackson, 1986; Bakker et al., 2002).

#### 4.1.3 Stage 3. Analysis of the correlation between grades by dimension and age

Once the degrees and levels of BS in the sample were analyzed, the relationship between the degree values reported in each dimension and the age of participants was determined by the index Spearman’s Rho. In this case, six age groups and ranges were studied: under 21 years old, 21-30, 31-40, 41-50, 51-60, and over 60 years. As a result, although the dimensions are highly correlated with each other, age was only significantly related to the dimension of cynicism (CC) (−0.084) and professional efficiency (PE) (0.069) with a significance level of 0.05. Accordingly, age exhibits negative and positive correlations with cynicism and professional efficiency, respectively, even when they are weak (see Table 7).

**TABLE 7:** Correlation between age and grades by dimension.

Dimension		Age	EE	CC
Cynicism (CC)	Correlation Coefficient	−0.084*	0.272^**^	1
	Sig. (bilateral)	0.01	0.001	
Professional efficiency (PE)	Correlation coefficient	0.069*	0.217^**^	−0.288^**^
	Sig. (bilateral)	0.036	0.001	0.001

*The correlation is significant at the 0.05 level (bilateral).

**The correlation is significant at the 0.01 level (bilateral).

In addition, the index Spearman’s Rho was recalculated to determine the correlation analysis between the BS degree value for each dimension and age. Unlike the first correlation analysis, the sample was divided into two groups: less than or equal to 40 and over 40 years (see Table 8). The results confirm the presence of a significant correlation between age among the three dimensions of BS, and age is significantly correlated with cynicism and the professional efficiency dimension, with a significance level of 0.01. Accordingly, age exhibits negative and positive correlations with cynicism and professional efficiency, respectively, even when they are weak.

**TABLE 8:** Correlation between age and BS grades by dimension.

Dimension		Age (40 years old)	EE	CC
Cynicism (CC)	Correlation coefficient	−0.094^**^	0.272^**^	1
	Sig. (bilateral)	0.004	0.001	
Professional efficiency (PE)	Correlation coefficient	0.098^**^	0.217^**^	−0.288^**^
	Sig. (bilateral)	0.003	0.001	0.001

**The correlation is significant at the 0.01 level (bilateral).

#### 4.1.4 Stage 4. Testing means between BS dimensions and age

So far, correlation analyses have shown significant relationships between cynicism grades and the dimensions of professional efficiency and age.

In addition, the Tukey test was performed to compare the means and identify the age groups with a significant difference. The BS level and the degrees of the three dimensions concerning the age range were analyzed. When analyzing the levels of BS, there were no significant differences between the ages studied. While analyzing by dimension, it was found that in emotional exhaustion, there were two significant differences between the means of the age groups: under 21 and from 31 to 40 years old; 21-30 and 31-40 years old. In both the cynicism and professional efficiency dimensions, significant mean differences were shown between the 21-30 and 31-40 age groups and those aged 51-60, finally, in the Professional Efficiency dimension (Table 9).

**TABLE 9:** Tukey test with α0.05.

Dependent variable	(I) What is your age	(J) What is your age	Mean difference (I-J)	Desv. Error	Sig.	95% confidence interval	
						Lower limit	Upper limit
Emotional exhaustion	31-40	Under 21	−0.771*	0.260	0.036	−1.51	−0.03
		21-30	−0.216*	0.068	0.021	−0.41	−0.02
Cynicism	51-60	21-30	−0.313*	0.109	0.046	−0.62	0.00
		31−40	−0.339*	0.108	0.021	−0.65	−0.03
Professional efficiency	51-60	21−30	0.339*	0.108	0.021	0.03	0.65
		31−40	0.440*	0.107	0.001	0.13	0.74

*The difference in means is significant at the 0.05 level.

## 5 Discussion

The results of our study expand the evidence regarding the necessity and importance of addressing BS as a multifactorial issue affecting employees across diverse professions and organizational levels (Lindblom et al., 2006; Armenta-Hernández et al., 2021; Singh et al., 2024). Likewise, it is observed that the correlation values in the few studies found in the literature between age and BS dimensions have also been weak, even with other study variables such as suicidal behaviors, turnover, and motivation, learning, among others (Lindblom et al., 2006; Ahola et al., 2008; de Jesus et al., 2023; Gómez et al., 2024; Singh et al., 2024; Lopes et al., 2025). Hence, these findings may make it challenging to design proper and effective interventions to diminish BS symptoms according to age.

Additionally, BS’s effects on industrial employees vary and can be severe depending on contextual, workplace, organizational, and individual factors. Among these, age is a critical variable that must be considered when studying and understanding the effects of BS and determining appropriate interventions and further research. The relationship between age and the dimensions of BS—emotional exhaustion, cynicism, and professional efficacy—has garnered significant attention in occupational health research, particularly in industrial settings. Recent studies have highlighted how age influences these dimensions, often interacting with other variables such as gender and workplace stressors. Furthermore, several of these studies, including ours, have contributed to confirming the validity of the 16-item Maslach BS Inventory (MBI-GS) across a wide range of samples, professions, and contexts.

Our findings provide a basis for comparison with relevant studies and contribute to a deeper understanding of the relationship between age and the dimensions of BS.

### 5.1 Age and emotional exhaustion

While the correlation analysis did not show significant correlations between emotional exhaustion and age, our findings align with previous studies, such as research conducted in a Moroccan private company, which demonstrated that younger workers (<35 years) exhibit higher levels of emotional exhaustion than their older counterparts. Specifically, our study revealed significant differences in emotional exhaustion between participants aged 3140 years and those under 21 years (*p* = 0.036), and the group aged 2130 years (*p* = 0.021). These results differ from the negative correlation between age and emotional exhaustion reported by other researchers (Macias-Velasquez et al., 2019), suggesting that older workers may develop more effective coping strategies or exhibit greater emotional resilience in response to occupational demands.

### 5.2 Age and cynicism

Significant correlations were obtained between age and cynicism (*r* = −0.084; *p* = 0.01) with a significance level of 0.05. However, although significant, the index is weak. This coincides with studies such as Flores Ortiz et al., 2016 and Posso-Yépez et al., 2025. Likewise, when the correlation analysis was performed with the sample divided into two age groups (less than or equal to 40 years and over 40 years), the significant correlation (*r* = −0.094; *p* = 0.004) was slightly increased with a significance level of 0.01.

Also, our findings are consistent with prior research indicating that cynicism tends to be more pronounced among younger workers, particularly those transitioning into industrial roles. We observed significant differences in cynicism between the 51-60 age group and the younger cohorts aged 21-30 years (*p* = 0.046) and 31-40 years (*p* = 0.021). These results align with the adaptive perspective highlighted in studies of middle managers in the maquiladora industry, where younger employees exhibited higher levels of cynicism as a response to workplace stressors and limited emotional resilience (Armenta-Hernández et al., 2021). Furthermore, our results support the negative correlation between age and cynicism (Armenta-Hernández et al., 2021; Arji et al., 2023), with younger workers often displaying higher levels of CC, reflected in correlation values ranging from −0.2 to −0.4. These findings underscore the importance of considering age-specific interventions to address cynicism and its underlying causes among younger industrial employees.

### 5.3 Age and professional efficacy

Significant correlations were obtained between age and professional efficiency (*r* = 0.069; *p* = 0.036) with a significant level of 0.05. However, although significant, the index is weak. This indicates that age is not a variable that determines the degree of the professional efficiency dimension. Similarly, when the correlation analysis was performed with the sample divided into two age groups (less than or equal to 40 years and over 40 years), the significant correlation (r = 0.098; p = 0.003) was slightly increased with a significance level of 0.01.

In addition, our results reveal significant differences in professional efficacy between the 51-60 age group and the younger cohorts aged 21-30 (*p* = 0.021) and 31-40 (*p* = 0.001), suggesting that professional efficacy varies substantially across age groups. Although older workers tend to report lower professional effectiveness, especially in sectors where technological change is occurring more rapidly, this decrease in reported effectiveness could be attributed to difficulties adapting to new technologies and changes in the demands to carry out the activities assigned to their jobs. This decrease in reported effectiveness could be attributed to difficulties adapting to new technologies and changes in demand levels to carry out the activities assigned to their job. In contrast, despite experiencing higher levels of BS, younger employees demonstrate greater professional effectiveness, probably due to being more familiar with new technologies and ways of integrating with modern workflows. These findings emphasize the need for targeted training programs for older employees to bridge technological skill gaps and sustain their professional efficacy in dynamic industrial environments.

### 5.4 The Maslach BS Inventory General Survey

The results regarding the 16-item Maslach BS Inventory General Survey (MBI-GS) provide strong support for its three-dimensional representation of BS and demonstrate good internal consistency, consistent with findings from prior studies (Adanaqué-Bravo et al., 2023; De Beer et al., 2024; Wang et al., 2024). The structure of the MBI-GS was validated using exploratory factor analysis (EFA), which showed good fit indices for the complete sample. However, when the sample was divided into age groups, the model’s fit indices did not meet the same standards, suggesting that these indicators are sensitive to sample size and that this sensitivity impacts the model’s performance across different age groups. Similar findings regarding cross-cultural occupational validity have been reported in other studies (Bakker and de Vries, 2021; Adanaqué-Bravo et al., 2023). Furthermore, our results align with those of Wang et al. (2024), which confirmed the overall reliability of the instrument and observed good fit indices both dimensionally and across age groups. However, further research is needed to use the MBI-GS across a broader range of professions to assess the consistency of its psychometric properties within the Mexican population. Future research may address these challenges to improve the applicability and accuracy of the instrument in diverse populations and age groups.

### 5.5 Future directions

Our research focused on analyzing how age interacts with the dimensions of BS in industrial settings. In line with this purpose, age can be an important moderating variable when making practical interventions tailored to a specific context. According to our results, younger workers could benefit from stress management programs and mentoring. In comparison, older workers should focus on continuous professional development to achieve and maintain suitability in technologically changing environments.

Future studies, building upon our research, should focus on:

1. Exploring longitudinal trends in the relationship between age and BS.
2. Validating BS measurement tools within more detailed demographic categories.
3. Investigating the moderating effects of other factors, such as gender, job role, and organizational culture, on age-BS dynamics.

## 6 Conclusion

The sample studied presents an average level of BS. When analyzing the BS levels, the sample distribution is similar in the five levels considered. The results reveal that, although the dimensions of BS are highly correlated, age only shows a significant relationship with the dimensions of cynicism and professional efficiency. In addition, the correlation analysis between the degree of BS of each dimension and age, categorized in two ranges: less than or equal to 40 years and greater than 40 years, confirms the existence of a significant correlation between age and the three dimensions of BS. In this analysis, age presents a significant correlation with the dimensions of cynicism and professional efficiency, with a significance level of 0.01, even when weak.

When examining the levels of BS, no significant differences were found between the ages studied. However, when analyzing each dimension separately, significant differences were found in emotional exhaustion between the age groups under 21 years and 31-40 years, and between the groups 21-30 and 31-40 years. In both the cynicism and professional efficacy dimensions, significant mean differences were observed between the 21-30 and 31-40 age groups and the 51-60 age groups. Accordingly, age exhibits negative and positive correlations with cynicism and professional efficiency, respectively, even when weak. Therefore, these findings may challenge the design of proper age-based interventions to diminish BS in the Mexican manufacturing industry employees and diminish its adverse effects.

### 6.1 Limitations of the study

As a limitation of the study, it is necessary to note that although significant effects were found between the correlations of the variables in this study, they are noticeably weak. This implies debate with other authors who found higher relationships between the study variables, but these are still weak relationships.

In addition, a considerable change in the parameters was observed since the sample was separated into two groups, up to and over 40 years of age. Referring to the model, those up to 40 years old do not comply with any absolute adjustment parameters. However, it complies with incremental fit parameters, which measure the degree to which the model’s fit is improved compared to the hypothetical model. This fact was considered enough statistical proof to compare age cohorts. Finally, the model with participants over 40 years of age only complies with one absolute fit parameter: the chi-square ratio for degrees of freedom, with a value less than 5.

Accordingly, few studies have yielded conclusive results on the relationship between BS dimensions and age. Similarly, studies that relate the dimensions of BS to other variables, such as suicidal tendencies, turnover, motivation, and learning, have also found weak correlations and moderating effects.

Additionally, this study acknowledges a limitation in the absence of control variables, such as gender, seniority in employment, sector, or educational level, within the sample. The interpretation of the age-BS relationship may be incomplete without these crucial variables. Their inclusion is necessary for a comprehensive understanding of the effect of age on the manifestation of BS in employees of the Mexican manufacturing industry. This limitation highlights the need for further research in this area.

## Data Availability

The original contributions presented in the study are included in the article, further inquiries can be directed to the corresponding author/s.
